# Antioxidant Activity of Medicinal Herbs and Spices from Plants of the Lamiaceae, Apiaceae and Asteraceae Families: Chemometric Interpretation of the Data

**DOI:** 10.3390/antiox12122039

**Published:** 2023-11-24

**Authors:** Beata Ulewicz-Magulska, Marek Wesolowski

**Affiliations:** Department of Analytical Chemistry, Medical University of Gdansk, Gen. J. Hallera 107, 80-416 Gdansk, Poland; beata.ulewicz-magulska@gumed.edu.pl

**Keywords:** medicinal herbs, spices, phenolic profile, antioxidant potential, Apiaceae, Asteraceae, Lamiaceae, PCA, HCA

## Abstract

Plant products, especially medicinal herbs and spices, have been used for centuries as a remedy to support human health and improve the flavor of food. Therefore, the purpose of this study was to identify plant species distinguished by their high content of phenolic compounds and high antioxidant activity using advanced multivariate statistical techniques such as Principal Component Analysis (PCA) and Hierarchical Cluster Analysis (HCA). To realize the purpose of the study, the total phenolic (TPC) and flavonoids (FC) content, antioxidant activity (TAC) and Fe(II) ion chelating capacity (FIC) of medicinal herbs and spices from plants belonging to three botanical families, Lamiaceae, Apiaceae and Asteraceae were determined. The interpretation of the obtained data revealed that the studied samples are localized in the PCA and HCA plots according to their TPC, FC, TAC and FIC values. Chemometric analysis confirmed that medicinal herbs and spices from plants belonging to the Lamiaceae family are richer sources of phenolic compounds and exhibit stronger antioxidant activity than those raw materials from plants in the Apiaceae family. In addition, no significant differences were found in terms of TPC, FC, TAC and FIC values between medicinal herbs and spices from the same plant species, i.e., oregano (*Origanum vulgare*), common thyme (*Thymus vulgaris*), rosemary (*Rosmarinus officinalis*), caraway (*Carum carvi*) and lovage (*Levisticum officinale*). A close relationship between antioxidant properties and contents of phenolic compounds was also confirmed.

## 1. Introduction

In the process of cellular oxygen reduction during ATP production, by-products known as reactive oxygen species (ROS) are formed in the human body. Among ROS, free radicals, such as OH^•^ or O^•^_2_, can be distinguished [1]. ROS can also come from exogenous sources such as smoking or environmental pollutants. The accumulation of ROS in the human body results in a redox imbalance. This leads to damage to many cellular structures, which can result in the disruption of cellular homeostasis [2,3]. Hence, when there is an imbalance between the ROS and endogenous antioxidants, exogenous antioxidants taken with food can be an effective factor in the fight against free radicals. Excellent alternative sources of natural antioxidants are plant products, such as fruits, vegetables and herbs [4,5,6,7]. They contain a number of substances with antioxidant activity, such as vitamins A, C and E and carotenoids [3,8]; phenolic compounds (phenolic acids, flavanols, flavones, catechins, anthocyanins and anthocyanidins, lignans and stilbenes); tannins; and coumarins [9].

Particular attention should be paid to phenolic compounds, which are the most abundant group of antioxidants in the plant world, as well as the most commonly ingested from food [10]. Phenolic compounds belong to a large group of secondary metabolites found only in the plant world. The basic element of their chemical structure is an aromatic ring substituted with hydroxyl groups. Therefore, they are classified into numerous groups due to their basic chemical structure and into subgroups depending on the number and position of hydroxyl groups and other substituents [11,12]. For this reason, among phenolic compounds, there are both simple and very complex compounds with a high degree of polymerization and high molecular weight [9,13]. The complexity of the chemical structure of phenolic compounds divides them into five groups, i.e., phenolic acids, flavonoids, stilbenes, lignans and tannins [14]. Phenolic compounds are characterized by biological activities, including antioxidant, anticancer, antiviral, anti-allergic and immune-stimulating activities. The antioxidant activity of phenolic compounds is due to their reducing properties, which allow them to act as a hydrogen donor, i.e., a reducing agent and scavenger of OH^•^ or O^•^_2_ radicals, as well as exhibit the ability to chelate transition metal ions [9]. These valuable properties of phenolic compounds are determined by the presence of numerous hydroxyl groups, from which hydrogen can be transferred to radicals, which are thus transformed into more stable and less reactive forms.

Medicinal herbs and spices have been valued since ancient times for their medicinal effects, as well as their flavor and aroma. The benefits arising from, among other things, the presence of antioxidants also make them widely used today, not only in medicine but also in cooking [15]. The biological activity of medicinal herbs and spices is influenced by the phenolic compounds, vitamins and trace elements present in them, as well as the volatile constituents of essential oils [16]. Substances of an antioxidant nature are most often found in plants in the form of complex mixtures, and their biological activity is often the result of synergism [12].

Taking into account the great importance of medicinal herbs and spices in the daily life of modern humans, the aim of the study was to identify, through chemometric analysis, plant species distinguished by their high content of phenolic compounds and high antioxidant activity and to determine the relationship between antioxidant properties and the content of phenolic compounds in order to confirm that phenolic compounds are responsible for the antioxidant properties of plants. In pursuing the goal of the study, we determined the total phenolic (TPC) and flavonoid (FC) contents of medicinal herbs and spices from plants belonging to three families, Lamiaceae, Apiaceae and Asteraceae, as well as their antioxidant activity, using DPPH and ABTS synthetic radicals and the FRAP (Ferric-Ion-Reducing Antioxidant Power) assay. In addition, the ferrous ions chelating capacity (FIC) by infusions of medicinal herbs and spices was also assessed. To achieve the research objective, advanced multivariate statistical techniques such as Principal Component Analysis (PCA) and Hierarchical Cluster Analysis (HCA) were used. Through multivariate reduction (PCA) and similarity search (HCA), both techniques facilitate the interpretation of multivariate experimental data sets. The literature offers examples of the use of PCA and HCA to study the relationship between antioxidant activity and phenolic profile of plant materials [17,18,19] to classify and discriminate medicinal plant materials based on antioxidant activity and chemical profile [6,20], or to compare methods used to determine antioxidant activity [21].

## 2. Materials and Methods

### 2.1. Plant Material

Eighty-seven plant raw materials from fourteen plant species belonging to three families, Lamiaceae, Apiaceae and Asteraceae, were selected for the study. The explored raw materials consisted of 39 samples of medicinal herbs and 48 samples of spices. The medicinal herbs and spices from the same five medicinal plant species constituted 40 samples. Table 1 shows the numbering of the raw materials studied, their commonly used names, and their intended use for medicinal (herbs) or culinary (spices) purposes. Information is also shown on the form in which they were in their original packages, as well as the systematic names of the plant species and botanical families from which the raw materials for the study came.

Medicinal herbs and spices were purchased from herbal and grocery stores in Gdansk (Poland). They came from 11 manufacturers located in Poland: Kotanyi (Warsaw), Kamis (Stefanowo), Dary Natury (Koryciny), Kawon (Gostyń), Flos (Mokrsko), Herbapol (Cracow), Appetita (Wykroty), Sigal (Wierzchosławice), Prymat (Jastrzębie Zdrój), Galeo (Stefanowo) and Cykoria (Wierzchosławice).

### 2.2. Preparation of Infusions

Medicinal herbs and spices were ground in a Knifetec Sample Mill 1905 (Foss Tecator, Höganäs, Sweden). A grain fraction of 80 to 250 µm was used for studying. Samples were placed in sealed plastic containers and stored in a dry and dark place until analysis.

Infusions of medicinal herbs and spices were prepared using procedures described in the literature [22,23]. Approximately 0.5 g of the sample, weighed on a WAA 100/X/1 analytical balance (Radwag, Radom, Poland), was poured over 25 mL of boiling redistilled water, stirred with a dipstick, covered with a watch glass and allowed to stand for 10 min. After cooling, it was filtered through MN 640 m, Ø 110 mm filter paper (Marcherey-Nagel, Düren, Germany) into volumetric flasks and diluted with redistilled water to a volume of 50 mL.

### 2.3. Reagents and Solutions

Reagents such as rutin trihydrate, ferrozine (3-(2-pyridyl)-5,6-di(2-furyl)-1,2,4-triazine-5′,5″-disulfonic acid disodium salt), trolox (6-hydroxy-2,5,7,8-tetramethyl chromane-2-carboxylic acid), ABTS (2,2′-azino-bis(3-ethylbenzothiazoline-6-sulfonic acid) diammonium salt), DPPH (2,2-diphenyl-1-picrylhydrazyl), TPTZ (2,4,6-tris (2-pyridyl)-s-triazine), gallic acid monohydrate, Folin and Ciocalteu’s phenol reagent and potassium persulfate were purchased from Sigma-Aldrich (Saint Louis, Mo, USA). Sodium carbonate anhydrous, iron(III) chloride hexahydrate, iron(II) sulfate heptahydrate, aluminum chloride hexahydrate, potassium acetate anhydrous, sodium acetate anhydrous, acetic acid 99.5–99.9%, methanol and ethanol 96% were obtained from POCH (Gliwice, Poland). Hydrochloric acid at 36.5–38% was acquired from (Baker Instra-Analyzed, Phillipsburg, NJ, USA). All chemicals and solvents were of analytical-grade reagents.

### 2.4. Determination of Total Phenolic Content (TPC)

A procedure using the Folin–Ciocalteu reagent was used to determine the TPC in the medicinal herbs and spices [24]. Due to the light sensitivity of the Folin–Ciocalteu reagent, the determinations were performed in centrifuge tubes shielded by aluminum foil.

To each tube, 4 mL of redistilled water, 1 mL of appropriately diluted infusion and 0.5 mL of Folin–Ciocalteu reagent were added. After 2 min, 2 mL of 20% sodium carbonate solution was added, and then the solutions in the tubes were diluted to 10 mL with redistilled water, mixed and incubated for 30 min in a dark place. Absorbance measurements were made using a UV-1202 spectrophotometer (Shimadzu, Duisburg, Germany) at 760 nm against a blank containing all reagents except the infusion. The TPC in the medicinal herbs and spices was expressed as Gallic Acid Equivalent (GAE) per gram of sample dry mass (mg GAE/g d.m.) using a calibration curve prepared from standard solutions of gallic acid at the concentration range of 1–8 µg/mL (Table 2).

### 2.5. Determination of Flavonoid Content (FC)

FC in the medicinal herbs and spices was determined using the ability of flavonoids to form stable, yellow-colored complexes with Al(III) ions. The color intensity of the complexes formed depends on the flavonoid content [25,26].

To each centrifuge tube, 0.5 or 2 mL of the infusion was added, depending on the type of raw material studied. Then, 1.2 mL of 0.1 mol/L methanolic aluminum chloride solution and 2 mL of 1 mol/L aqueous potassium acetate solution were added. The tubes were supplemented with 75% ethanol solution to a volume of 10 mL, mixed thoroughly and incubated for 40 min in a dark place. Absorbance was measured at 415 nm against a blank sample containing all reagents except the infusion. The assay result was calculated using a calibration curve prepared from standard solutions of rutin at a concentration range of 4.0–28.0 µg/mL (Table 2). FC in medicinal herbs and spices was expressed as rutin equivalent (RUT) per gram of sample dry mass (mg RUT/g d.m.).

### 2.6. Determination of Total Antioxidant Capacity (TAC)

The TAC of medicinal herb and spice infusions was determined using three assays using the organic synthetic radical DPPH, the ABTS radical cation, and the FRAP method.

#### 2.6.1. DPPH Assay

In this assay, antioxidants reduce the alcoholic solution of violet-colored DPPH, resulting in a change in the color to yellow. The antioxidant potential reflects the amount of DPPH radical that was reduced by the antioxidants in the sample [27,28,29].

To each centrifuge tube, protected from light by aluminum foil, 0.3 mL of diluted infusion and 4 mL of methanolic DPPH solution were added. After incubation in the dark for 10 min, the absorbance at 517 nm was measured against a blank sample containing all reagents except the infusion. The antioxidant potential of medicinal herb and spice infusions was expressed as the percentage of DPPH radical inhibition calculated according to the equation:

(1) $$Inhibition\;\left(\%\right)=\frac{A_{0}-{\;A}_{infusion}}{A_{0}\;\cdot \;100}$$

*A*_0_—absorbance of the blank sample; *A_infusion_*—absorbance of the studied sample.

#### 2.6.2. ABTS Assay

The ABTS assay involves the generation and subsequent deactivation of the ABTS^•+^ radical cation [30,31]. The ABTS^•+^ solution was prepared by mixing aqueous solutions of ABTS at 7 mmol/L and potassium persulfate at 2.45 mmol/L. Before use, the solution was diluted with 96% ethanol to obtain absorbance in the range of 0.7–0.8 at 734 nm.

To each centrifuge tube covered with aluminum foil, 100 µL of the diluted infusion and 3.9 mL of alcoholic solution of ABTS^•+^ radical cation were added. After incubation in a dark place for 10 min, the absorbance of the solutions was measured at 734 nm against a blank containing 3.9 mL of ethanolic solution of ABTS^•+^ and 100 µL of redistilled water instead of the infusion. A calibration curve was prepared using standard solutions of trolox in the concentration range of 0.1–1.0 µmol/mL (Table 2). The antioxidant activity of the trolox solutions and the studied infusions was expressed as the percentage of inhibition of ABTS^•+^ activity, calculated from Equation (1). Based on the calibration curve showing the percentage of inhibition of ABTS^•+^ activity as a function of the trolox concentration, the antioxidant activity of medicinal herbs and spices was calculated as Trolox Equivalent Antioxidant Capacity (TEAC) per gram of sample dry mass (mmol TEAC/g d.m.).

#### 2.6.3. FRAP Assay

The FRAP assay measures the ability of antioxidants to reduce Fe(III) ions [32,33,34]. The FRAP solution was prepared by mixing in a volume ratio of 10:1:1 the following solutions: acetate buffer at 300 mmol/L (pH = 3.6), iron(III) chloride at 20 mmol/L and TPTZ at 10 mmol/L in 40 mmol/L HCl.

To each centrifuge tube, 0.1 mL of infusion or suitably diluted infusion, 1.9 mL of FRAP solution was added and, after topping up to 10 mL with redistilled water, incubated on a water bath at 37 °C for 10 min. The absorbance of the solutions was then measured at 593 nm relative to a blank prepared in the same manner as the studied solutions, with redistilled water instead of the infusion. The result of the determination was calculated using a calibration curve prepared from standard solutions of iron(II) sulfate at the concentration range of 2.5–40 µmol/L (Table 2). The antioxidant potential of infusions of medicinal herbs and spices was expressed in mmol Fe(II)/g d.m.

### 2.7. Determination of Ferrous Ions Chelating Capacity (FIC)

Flavonoids are capable of forming complexes with iron(II) ions [35]. Excess Fe(II) cations not bound by flavonoids are bound into a colored complex with ferrozine, resulting in a color change in the solution from yellow to blue. The intensity of the blue color is greater the more iron(II) ions are complexed by ferrozine.

To a test tube, 1 mL of the infusion was measured, 100 µL of 2 mmol/L iron(II) sulfate solution was added, and they were mixed very thoroughly and allowed to stand for 2 min. Then, 100 µL of 5 mmol/L ferrozine solution was added to each tube, diluted with redistilled water to a volume of 4 mL and mixed, and incubated at room temperature. After 10 min of incubation, the absorbance at 562 nm was measured against a blank sample containing all reagents except the infusion. The ability of antioxidants to chelate metal ions was expressed as the percentage of inhibition of ferrozine–Fe(II) complex activity calculated according to Equation (1).

### 2.8. Statistical Analysis

All measurements were taken in triplicate, and results are presented as arithmetic mean ± standard deviation (SD). Two advanced multivariate statistical techniques, Principal Component Analysis (PCA) and Hierarchical Cluster Analysis (HCA), were used to extract the maximum useful information from a large experimental data set [36]. For PCA and HCA calculations, a matrix of size n · p was constructed, in which the rows (n) were medicinal herbs and spices numbered 1–22 and 61–78 (objects) and columns (p) were data on the content of phenolic compounds and flavonoids, antioxidant activity and chelating capacity of Fe(II) ions (variables). Calculations were performed using Statistica StatSoft Inc. software, version 13.3 (Tulsa, OK, USA).

PCA uses mathematical calculations that convert a large number of correlated variables (experimental data) into a smaller number of uncorrelated (orthogonal) variables, called principal components (PCs). These new variables describe the variation in the experimental data. The first principal component (PC1) encompasses the greatest variability in the experimental data, the second principal component (PC2) describes the next greatest variability, and so on. Hence, the two-dimensional plot, PC1 and PC2, illustrates the classification of the samples under study taking into account the experimental data, and samples with similar properties are grouped together in the same cluster.

Unlike PCA, HCA involves looking for similarities and dissimilarities between objects (samples) and, on this basis, classifying studied samples into groups with similar properties. HCA calculations use the Euclidean distance as a measure of similarity between objects in a multidimensional space. In turn, Ward’s algorithm was used to group objects into clusters. In turn, the number of statistically significant clusters was extracted using Sneath’s index at 33% of the maximum distance. Therefore, clusters below 33% of the maximum distance are characterized by high mutual similarity.

## 3. Results and Discussion

The study explored infusions obtained from 87 samples of medicinal herbs and spices from 14 plant species belonging to three families: the Lamiaceae, Apiaceae and Asteraceae. The study is a continuation of previous work [37], in which only TPC and TAC were determined using two assays (DPPH and FRAP) in the methanol and water extracts. In this work, a much broader study was conducted, determining TPC, FC and TAC using three assays (DPPH, FRAP and ABTS) and FIC in the infusions. Infusions were chosen because they are a very common way of preparing herbs for consumption. They are prepared in the same way that tea is brewed. Thus, the results obtained for infusions reflect the amount of phenolic compounds and their oxidative activity that people realistically use when using medicinal herbs or spices.

Many plant species under study are used in medicine and as spices [38]. Medicinal herbs must be characterized using high-quality standards, including, among other things, the content of biologically active compounds. Detailed requirements are specified in the European Pharmacopoeia [39] or National Pharmacopoeias [40]. Spices, on the other hand, which are used as additives to food products and dishes to prolong their shelf life or improve their flavor, are controlled only for the presence of heavy metal contaminants [41]. Therefore, it also seems interesting to compare the results of the TPC, FC, TAC and FIC of medicinal herbs and spices from the same plant species. Multivariate methods of statistical analysis may play a particularly useful role in such a comparison [36].

### 3.1. Content of Phenolic Compounds

Phenolic compounds found in plants play an important role in stabilizing proteins, lipids and DNA, protecting them from oxidation. They take part in the scavenging of free radicals by inhibiting enzymes responsible for the production of reactive oxygen species, counteract the breakdown of peroxides into free radicals and convert radicals into less reactive forms [42,43,44].

The largest group of raw materials examined were samples of plants from the Lamiaceae family, viz. oregano (*Origanum vulgare*), common thyme (*Thymus vulgaris*), rosemary (*Rosmarinus officinalis*), lemon balm (*Melissa officinalis*), peppermint (*Mentha piperita*), common sage (*Salvia officinalis*), summer savory (*Satureja hortensji*), hyssop (*Hyssopus officinalis*), basil (*Ocimum basilicum*) and marjoram (*Origanum majorana*). Plants in the Lamiaceae family are considered a rich source of phenolic compounds with high biological activity, comprising mainly phenolic acids such as caffeic and rosmarinic acids and flavonoids in the form of ester derivatives or glycosides [45].

Besides caffeic and rosmarinic acids, other cinnamic acid derivatives such as ferulic, *p*-hydroxycinnamic, *p*-coumaric and chlorogenic acids are also present in the raw materials examined [46]. Data from the literature revealed that the content of these acids varies in the medicinal herbs and spices. Differences occur even within the same plant species and are caused by the environmental conditions in which the plant grew, the morphological part explored and the analytical method used for determination. The content of cinnamic acid derivatives in culinary herbs has been found to decrease in the following order: rosmarinic acid > caffeic acid > *p*-coumaric acid > ferulic acid > chlorogenic acid [16]. For example, the contents of rosmarinic, caffeic and chlorogenic acids are 156.90, 12.58 and 1.07 µg/g d.m. in rosemary and 52.02, 10.60 and 0.71 µg/g d.m. in oregano, respectively. On the other hand, the average TPC and TAC values (TAC determined using tDPPH and ABTS assays and expressed as Trolox Equivalent, TE) are 5.02 mg GAE/g d.m., 1.98 mmol TE/g d.m. and 2.39 mmol TE/g d.m. for rosemary and 2.23 mg GAE/g d.m., 0.78 mmol TE/g d.m. and 1.34 mmol TE/g d.m. for oregano, respectively.

The presence of hydroxyl groups linked to the aromatic ring affects the antioxidant properties of cinnamic acid derivatives [46]. Studies have shown that an increase in the number of hydroxyl groups in the *ortho* and *para* positions of the aromatic ring probably increases the antioxidant properties of phenolic acids [47]. For example, the TAC determined using the DPPH assay is 44.0 ± 0.5 and 93 ± 4% for caffeic and chlorogenic acids, respectively. Under in vivo conditions, the antioxidant potential of phenolic acids depends additionally on their bioavailability. The better the bioavailability of phenolic compounds, the higher the concentration of their metabolites in target tissues and the higher the antioxidant potential [48,49]. Among phenolic compounds, isoflavones and gallic acid are the most strongly absorbed by the human body, while anthocyanins are the least absorbed [49]. On the other hand, limited data on cinnamic acid derivatives make it impossible to determine bioavailability and reliably assess the antioxidant activity of these acids in relation to other phenolic compounds.

For the determination of total phenolic compounds (TPCs), the Folin–Ciocalteu reagent was used. This is a commonly used procedure involving the transfer of electrons from phenolic compounds to the Folin–Ciocalteu reagent in an alkaline medium. However, it should be noted that other compounds (sugars, ascorbic acid and proteins) can also give a response when reacted with this reagent [42]. The data gathered in Table 3 indicate that medicinal herbs and spices from plants belonging to the Lamiaceae family are the richest source of phenolic compounds. The average content of TPC in most samples exceeds 40 mg GAE/g d.m. Exceptionally high levels of phenolic compounds were found in lemon balm leaves (87.54 mg GAE/g d.m.), oregano herbs (82.66 mg GA/g d.m.) and oregano spice (54.30 mg GAE/g d.m.). In contrast, sage leaves (33.84 mg GAE/g d.m.) and spices, basil (33.65 mg GA/g d.m.) and savory (35.97 mg GAE/g d.m.) are the poorest in phenolic compounds.

Medicinal herbs and spices from plants belonging to the Apiaceae family, such as caraway (*Carum carvi*), lovage (*Levisticum officinale*) and garden angelica (*Archangelica officinalis*), on the other hand, are distinguished by their low content of phenolic compounds, below 10 mg GAE/g d.m. Only lovage spice has a higher TPC content (16.71 mg GAE/g d.m.) than the medicinal raw material, lovage root (6.28 mg GAE/g d.m.) and other samples from this family. Only one spice, tarragon, from the Asteraceae family is characterized by a TPC value of 32.35 mg GAE/g d.m.

The data specified in Table 3 also indicate that, as in the case of TPC, the average flavonoid content (TF) of medicinal herbs and spices was higher for samples from plants in the Lamiaceae family than from plants in the Apiaceae family. The richest in flavonoids are peppermint leaves (27.18 mg RUT/g d.m.), oregano herbs (25.40 mg RUT/g d.m.) and thyme herbs (21.16 mg RUT/g d.m.). Tarragon spice, from the Asteraceae family, is characterized by a TF value of 20.24 mg RUT/g d.m.

**Figure 1:**
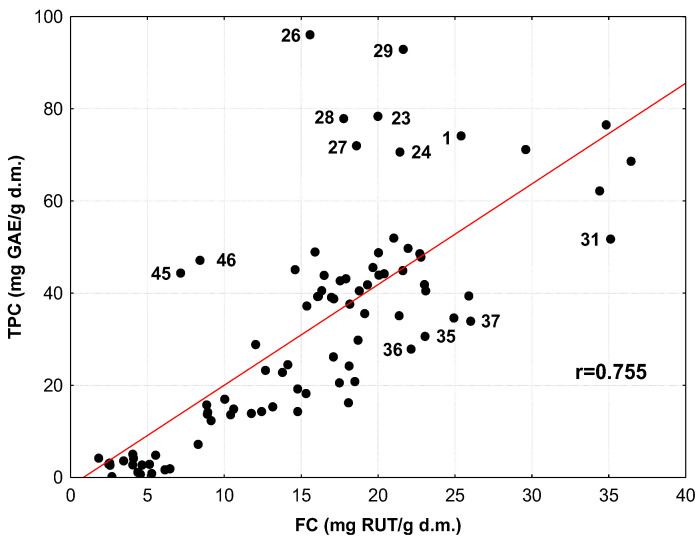
Relationship between total phenolic compounds (TPCs) and flavonoid content (FC) in infusions of the studied medicinal herbs and spices. Infusions distinctly deviating from the rectilinear relationship are marked with Arabic numerals, according to the numbering of the samples in the first column of Table 1 and Table 3.

The relationship between TPC and FC in the medicinal herbs and spices is shown in Figure 1. An analysis of this plot and the value of the correlation coefficient (r = 0.755, *p* < 0.001) exhibit a strong relationship between TPC and FC values. Only a dozen samples clearly deviate from this relationship. For example, these are oregano herbs (sample 1, according to the numbering of samples in the first column of Table 1 and Table 3); leaves of lemon balm (23, 24, 26–29), peppermint (31, 35) and sage (36, 37); and hyssop spices (45, 46). Based on the data listed in Table 3, it can be assumed that those of the aforementioned samples located in Figure 1 above the regression line (1, 23, 24, 26–29, 45, 46) have relatively low FC values in relation to the phenolic compound content (TPC) of the medicinal herbs and spices. In contrast, the samples in Figure 1 below the regression line (31, 35, 36, 37) contain significantly more flavonoids than the other samples containing similar amounts of phenolic compounds (TPCs).

A strong relationship between TPC and FC values is also indicated by data from the literature for infusions of Thai plants (r = 0.92) [42] and leaves of various apple species (r = 0.84–0.98) [50]. However, it should be noted that extracts with a high content of phenolic compounds are not always distinguished by a high concentration of flavonoids, since, depending on the plant species and morphological part, flavonoids can account for a different proportion of the total content of phenolic compounds [42].

### 3.2. Antioxidant Activity

The activity of natural antioxidants is associated with a number of mechanisms, so various assays have been developed to measure these properties in vitro. Some assays are based on examining the ability of antioxidants to inhibit lipid or lipoprotein oxidation, while others are based on activity toward free radical elimination. In addition, the antioxidant properties of plant infusions are the result of the synergistic action of all the active constituents contained in them. Therefore, the antioxidant potential of plant material can only be reliably assessed based on the results obtained via several different assays.

Three assays based on different mechanisms were chosen to evaluate the antioxidant activity of infusions of medicinal herbs and spices. The DPPH and ABTS assays are used to measure the antioxidant activity of compounds that are electron and/or hydrogen atom donors, while the FRAP assay assesses the antioxidant activity of substances that provide an electron [51,52]. These are fast, sensitive and widely used assays for assessing the antioxidant potential of both single compounds and chemically complex material, i.e., plant infusions, juices, wines and animal tissues [53].

The results obtained using three different assays (DPPH, ABTS and FRAP) for 87 infusions of medicinal herbs and spices from plants belonging to three botanical families (Lamiaceae, Apiceae and Asteraceae) are summarized in Table 3. A general interpretation of these data indicates that the highest antioxidant potentials are found in medicinal herbs and spices characterized by high TPC and FC values. These include samples of plants from the Lamiaceae and Asteracea families, while raw materials from the Apiaceae family are characterized by low levels of antioxidant activity, corresponding to low contents of phenolic compounds.

An assay using the DPPH radical was first described by Blois in 1958 [23], and since then, after numerous modifications, it has been widely used to evaluate the antioxidant properties of plant material. DPPH is a stable dark purple radical that reacts with other radicals (R^•^) or antioxidants (AH), i.e., reducing agents that provide a hydrogen atom [3,27,28]:

DPPH^•^ + R^•^ = DPPH–R

DPPH^•^ + AH = DPPH–H + A^•^

The data compiled in Table 3 indicate that the total antioxidant capacity of the examined infusions as determined using the DPPH assay (TAC_DPPH_) ranged from 10.72 to 92.87%. They also show that medicinal herbs and spices from plants belonging to the Lamiaceae family exhibit strong antioxidant properties. In addition, the highest TAC_DPPH_ values were shown by infusions that also had a high content of phenolic compounds determined. On the basis of antioxidant activity, the examined samples were arranged in the following order: lemon balm leaves > oregano herbs > oregano > peppermint leaves > hyssop > thyme > marjoram > rosemary leaves > thyme herbs > rosemary > savory > sage leaves > basil. The lowest antioxidant activity was shown by samples also characterized by a low content of phenolic compounds (TPCs). These were medicinal herbs and spices from plants in the Apiaceae family, such as caraway seeds and caraway, lovage root and lovage, and angelica root, as well as the spice from the Asteraceae family, tarragon. The activity of these raw materials was at the level of several percent.

To evaluate the antioxidant activity of solutions of substances, infusions of plants, or beverages, an assay using the synthetic ABTS^•+^ radical cation is also widely used. The original assay is based on the formation of a ferricmyoglobin radical from hydrogen-peroxide-activated methmyoglobin in the presence of the synthetic substrate ABTS [30,54]. The radical formed in this reaction generates the formation of the ABTS^•+^ radical cation. In the modified method, potassium or sodium persulfate is usually used to obtain the active radical cation [9]. The principle of the determination using this assay is the reduction of the ABTS^•+^ radical cation by antioxidants to a degree that depends on the concentration of the antioxidant, its activity and the time of the reaction. The decrease in the color intensity of the solution is proportional to the antioxidant content [9,30]. The good solubility of the radical cation in water and organic solvents allows the TPC determination of hydro- and lyophilic compounds [44,50].

The data compiled in Table 3 indicate that the TAC_ABTS_ of the examined infusions of medicinal herbs and spices ranges from 0.17 to 7.81 mmol TEAC/g d.m. The herbs lemon balm and oregano have the highest antioxidant activity. Considering TAC_ABTS_ values, infusions from plants of the Lamiaceae family can be ranked in the following order: lemon balm leaves > oregano herbs > marjoram > rosemary > oregano > rosemary leaves > peppermint leaves > thyme herbs > hyssop > thyme > sage leaves > basil > savory. As with TAC_DPPH_, infusions obtained from plant species belonging to the Apiaceae family also show the lowest TAC_ABTS_ values. Therefore, the antioxidant activity of infusions from caraway and lovage (medicinal herb and spice), angelica root (medicinal herb) and tarragon (spice, Asteraceae) ranges from 0.17 to 2.24 mmol TEAC/g d.m.

The principle of the FRAP determination is the reduction, in the presence of antioxidants, in an acidic medium, of the colorless Fe(III)–TPTZ complex to the Fe(II)–TPTZ complex, with a blue color measured at 593 nm.

Based on the data listed in Table 3, it can be concluded that TAC_FRAP_ values for infusions of medicinal herbs and spices range from 0.03 to 2.64 mmol Fe(II)/g d.m. Infusions of lemon balm leaves and thyme spice, as well as infusions of oregano herbs and oregano spice, showed the strongest antioxidant properties. They are also characterized by a high content of phenolic compounds and high antioxidant capacity, as determined by TAC_DPPH_ and TAC_ABTS_ assays. Literature data confirm that the leaves of plants, especially those of the Lamiaceae family, i.e., oregano, common thyme, rosemary, and basil, show strong antioxidant activity, in which the rosmarinic acid present in these plants has a large share [55,56]. Therefore, they are used, among other things, as a source of antioxidants in the food-production process.

To explore the extent to which the antioxidant activity of medicinal herb and spice infusions depends on the total phenolic compound (TPC) content, the data for TAC_DPPH_, TAC_ABTS_ and TAC_FRAP_ were plotted as a function of TPC. The strongest correlation was found in the DPPH results (r = 0.963, *p* < 0.001). Similar correlations were detected in infusions of Argentine herbs (r = 0.857) [57], coffees (r = 0.922) [58] and medicinal plants from India (r = 0.938) [59], and ethanol–water extracts of selected medicinal plants (r = 0.966) [60]. A slightly weaker correlation was found between antioxidant activity as determined by ABTS and TPC (r = 0.941, *p* < 0.01). Literature data also confirm the correlation between these values for green, black and white tea infusions and lemon balm and peppermint (r = 0.975–0.985) [61], apple leaf infusions (r = 0.84–0.98) [50] and Polish medicinal plants (r = 0.957) [60], (r = 0.964) [62]. TAC_FRAP_ values correlate slightly less strongly with TPC (r = 0.879, *p* < 0.000001). The literature also confirm a close correlation between the ability to reduce Fe(III) ions and the total content of phenolic compounds in infusions of medicinal plants used in Europe, Argentina and India (r ranging from 0.81 to 0.99) [53,57,59] and ethanol–water extracts of selected medicinal plants (r = 0.903) [60].

The results indicate that antioxidants present in medicinal herbs and spices are capable of eliminating free radicals and reducing oxidants. Strong correlations between antioxidant activity determined through DPPH, ABTS and FRAP assays and the total content of phenolic compounds confirm that these compounds, especially phenolic acids and flavonoids [13,63], contribute significantly to the antioxidant properties of plants. It should also be mentioned that there is sometimes contradictory information in the literature about the relationship between TPC and TAC. Some authors have found a strong relationship between these values [50,61,62], while others have not found it or have shown only a very weak relationship [43,44,64,65]. The contradictory data may be due to different extraction methods or different assays for determining antioxidant capacity. Also, other compounds present in plant infusions, i.e., proteins, vitamins and carbohydrates, contribute to the antioxidant properties of the studied material. They can also affect the results of TAC and TPC determinations when the Folin–Ciocalteu reagent is used [43,65,66].

**Figure 2:**
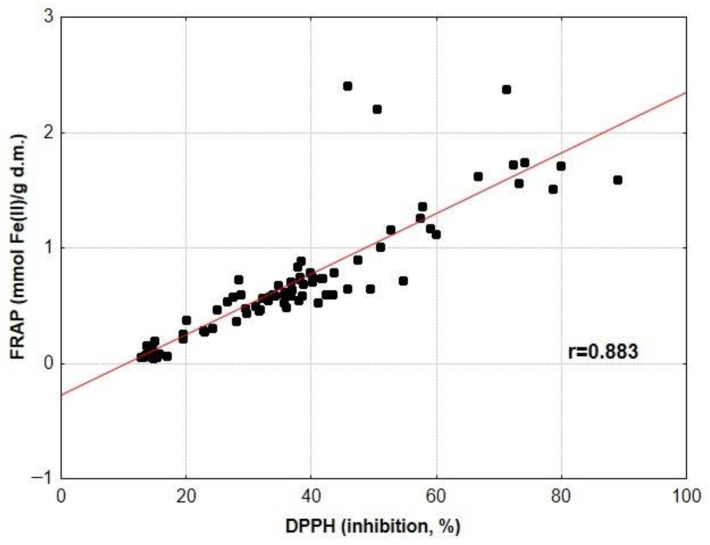
Relationship between values of TAC_FRAP_ and TAC_DPPH_ for infusions of medicinal herbs and spices.

**Figure 3:**
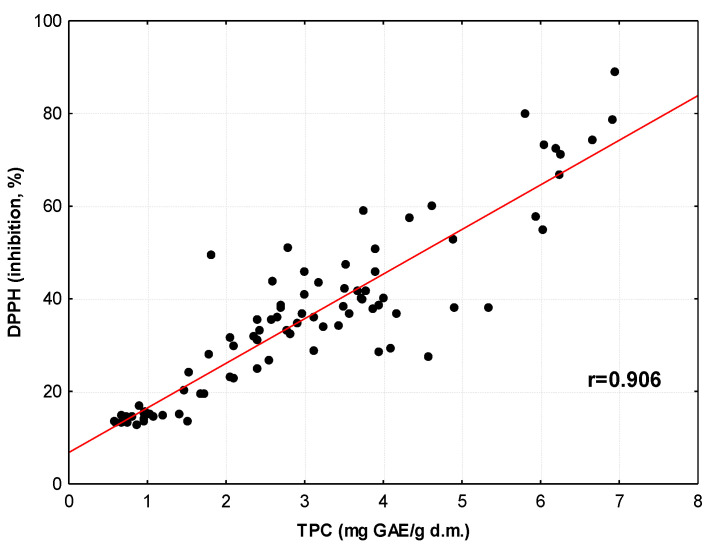
Relationship between values of TAC_DPPH_ and TAC_ABTS_ for infusions of medicinal herbs and spices.

**Figure 4:**
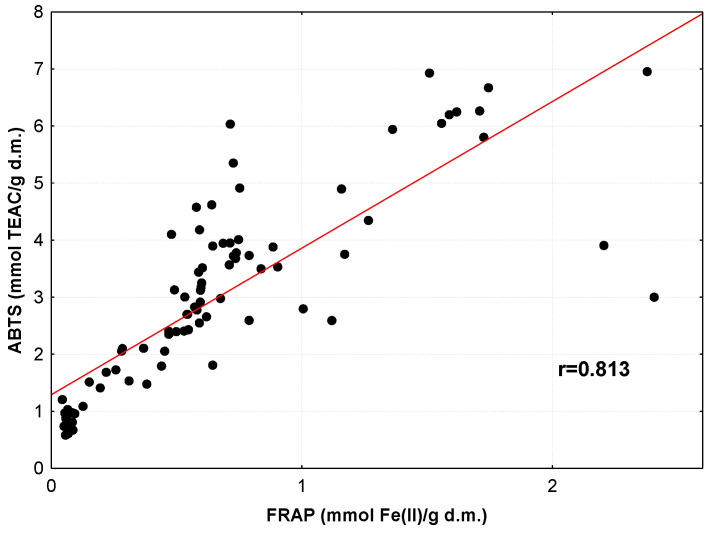
Relationship between values of TAC_ABTS_ and TAC_FRAP_ for infusions of medicinal herbs and spices.

Because the antioxidant activity of medicinal herbs and spices was explored using three different assays (DPPH, ABTS and FRAP), we decided to examine the relationship between the results generated by these assays. This is graphically illustrated in Figure 2, Figure 3 and Figure 4. Analyses of these plots and the values of the correlation coefficients indicate the strong ability of plant antioxidants to both inhibit the activity of DPPH and ABTS radicals and to reduce Fe(III) ions. Similar relationships between TAC_DPPH_ and TAC_FRAP_ values were detected for yellow soybean infusions (r = 0.822) [67], Serbian medicinal plants (r = 0.908) [52] and Indian medicinal plants (r = 0.881) [59]. High correlation coefficients between TAC_DPPH_, TAC_ABTS_ and TAC_FRAP_ values were also found for ethanol–water extracts of selected medicinal plants (r = 0.819–0.972) [60].

### 3.3. Ferrous Ions Chelating

Fe(II) ions play an important role in catalyzing the decomposition of hydrogen peroxide or lipid peroxides, resulting in the formation of hydroxyl radical (OH^•^), according to the Fenton reaction:

Fe^2+^ + H_2_O_2_ = Fe^3+^ + OH^−^ + OH^•^

Fe(III) ions can also participate in catalyzing the reactions leading to the formation of radicals, but their reactivity is 10 times lower than that of Fe(II) ions [35]. The chelation of Fe(II) ions therefore leads to inhibition of the Fenton reaction and prevents the formation of reactive hydroxyl radicals responsible for oxidative damage to lipids, proteins and DNA.

Ferrous ions chelating capacity (FIC) of infusions indicates how effectively the compounds in them compete with ferrozine in forming complexes with Fe(II). In the presence of chelating agents, the formation of the ferrozine–Fe(II) complex is disrupted, resulting in a weaker blue coloration of the solution. Measuring the absorbance of this solution allows estimating the chelating activity of the studied material [68,69]. The ability to chelate Fe(II) ions is demonstrated by biologically active substances present in plant infusions, including phenolic compounds, especially flavonoids [69,70,71].

Based on the data gathered in Table 3, the chelating capacity of Fe(II) ions by phenolic compounds present in infusions of medicinal herbs and spices ranged from 4.95 to 80.59%. Savory, sage leaves, basil, lemon balm leaves, thyme herbs, and peppermint leaves show the highest chelating capacity.

An exploration of the relationship between the content of phenolic compounds and flavonoids and the chelating capacity of Fe(II) ions by infusions of the medicinal herbs and spices studied indicated very weak relationships between TPC and FIC (r = 0.286, *p* < 0.01) and between FC and FIC (r = 0.410, *p* < 0.001). The lack of a statistically significant correlation between these data was also described in the literature for infusions of medicinal plants and spices [70]. The results indicate that other compounds in plant infusions are also capable of binding Fe(II) ions.

### 3.4. Multivariate Analysis

**Figure 5:**
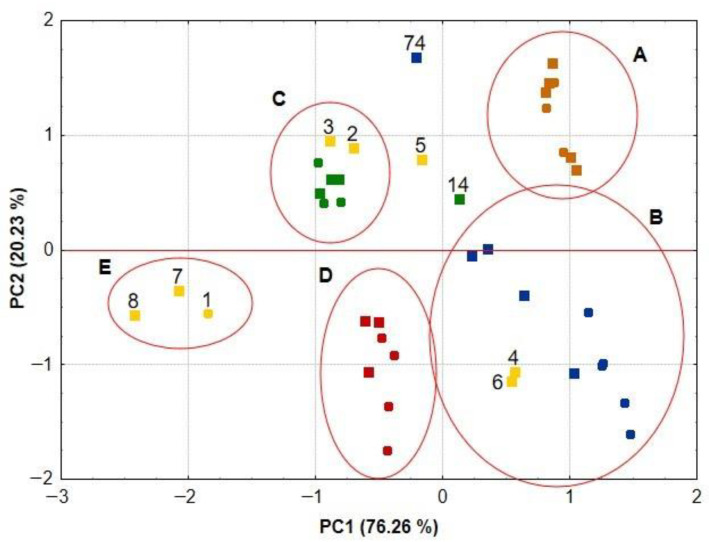
PCA score scatterplot for the medicinal herbs and spices originated from the same species of medicinal plants: ● oregano herbs, ■ oregano, ● thyme herbs, ■ thyme, ● rosemary leaves, ■ rosemary, ● caraway seeds, ■ caraway, ● lovage root and ■ lovage.

To identify the differences between medicinal herbs and spices, on one hand, and the belonging of the samples explored to a particular botanic family of medicinal plants, on the other hand, Principal Component Analysis (PCA) and Hierarchical Cluster Analysis (HCA) were used [36]. The data compiled in Table 3, i.e., total phenolic content, flavonoids content, total antioxidant capacity and chelating capacity of Fe(II) ions, were used to calculate PCA and HCA. Only those medicinal herbs and spices that originated from the same plant species were subjected to chemometric analysis. Therefore, the data matrix included five medicinal plant species, i.e., 40 samples of medicinal herbs and spices numbered 1–22 and 61–78 (according to the numbering of the samples in the first column of Table 1 and Table 3). These include oregano (*Origanum vulgare*), common thyme (*Thymus vulgaris*), rosemary (*Rosmarinus officinalis*), caraway (*Carum carvi*) and lovage (*Levisticum officinale*). The results of the PCA calculations are shown in Figure 5.

Figure 5 illustrates the distribution of samples in the two-dimensional plot of PC1 and PC2. The first two PCs together explain more than 96% of the variability studied. By analyzing this plot, it can be seen that most of the medicinal herbs and spices from plants of the same species were found in the same clusters. Cluster A groups all caraway samples (seeds and spices), the poorest in phenolic compounds, flavonoids and distinguished by the weakest antioxidant activity, with a significant ability to chelate Fe(II) ions. In the lower right corner (cluster B) are lovage (root and spices) samples, which were also distinguished by low TPC, FC, TAC and FIC values and, like caraway, came from a medicinal plant belonging to the Apiaceae family. Clusters C, D and E formed samples of medicinal herbs and spices from the Lamiaceae family. Rosemary samples (leaves and spices) are located in cluster D, while thyme samples (herbs and spices) are located in cluster C, with the exception of sample 14. They are characterized by phenolic compound contents of more than 40 mg GAE/g d.m. and average antioxidant activity among the tested material.

The group of raw materials most diverse in terms of TPC, FC and TAC values were spices of oregano (2–8, according to the numbering of the samples in the first column of Table 1 and Table 3). As shown in Figure 5, in cluster E, there were only two spices of oregano (7, 8) and herb of oregano (1). These samples were characterized by a very high content of phenolic compounds and flavonoids, as well as high antioxidant activity and chelating capacity. Two more spices of oregano (2, 3) group together with herbs and spices of thyme in cluster C. The reason for this location is the lower TPC, FC and TAC values for the oregano samples compared to the samples located in cluster E. In contrast, samples 4 and 6, characterized by the lowest content of phenolic compounds, flavonoids and low antioxidant activity and chelating capacity, are located in cluster B. The last spice of oregano (5), together with spices of thyme (14) and lovage (74), is located in the area between clusters A and C. This location was determined by the high FIC values characterizing the three spices at the same time as low TPC, FC and TAC values.

The interpretation of the PCA results showed that the studied medicinal herbs and spices localize along the PC1 axis, consistent with changing values of TPC, FC, TAC and FIC. Moving from the right to the left side of the PCA plot, the content of phenolic compounds and antioxidant activity increases gradually, reaching the highest values for samples located on the left side of the PCA plot. This principle is not always followed by FIC values. It happens that samples with a high ability to chelate Fe(II) ions are located on both the left and right sides of the PCA plot. The PCA calculations also confirm previous observations that medicinal herbs and spices from plants belonging to the Lamiaceae family, located in clusters C, D and E, are richer sources of phenolic compounds and exhibit stronger antioxidant activity than medicinal herbs and spices from plants belonging to the Apiaceae family, located in clusters A and B. In addition, there were no significant differences in terms of TPC, FC, TAC and FIC values between medicinal herbs and spices from the same plant species.

**Figure 6:**
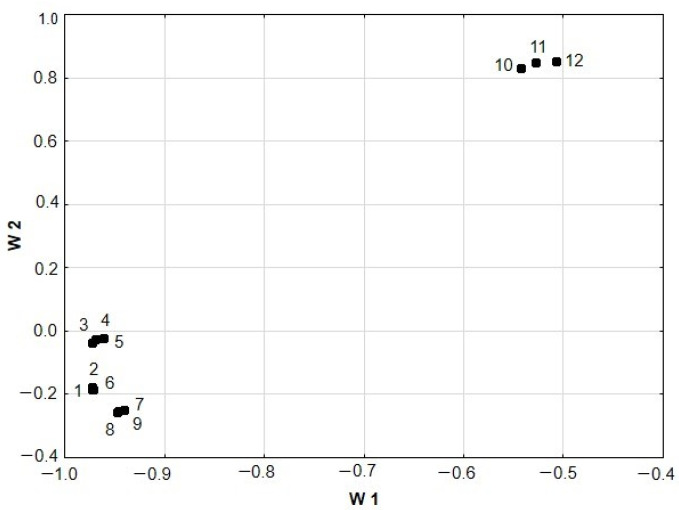
Two-dimensional loading plot of W1 and W2 calculated for the medicinal herbs and spices originating from the same species of medicinal plants. Variables: 1—TPC min, 2—TPC max, 3—FC min, 4—FC max, 5—DPPH min, 6—DPPH max, 7—ABTS min, 8—ABTS max, 9—FRAP min, 10—FRAP max, 11—FIC min, and 12—FIC max.

Figure 6 shows a plot of loadings (W1 and W2) for the first two PCs. An analysis of this plot indicates that the distribution of medicinal herbs and spices along the PC1 axis is determined by the content of phenolic compounds and flavonoids (variables 1–4) and the antioxidant potential determined by the DPPH, ABTS and partially FRAP assays (variables 5–9). In turn, the chelating capacity of Fe(II) ions and partially antioxidant potential determined by the FRAP assay (variables 11–12) determines the distribution of the explored samples along the PC2 axis.

**Figure 7:**
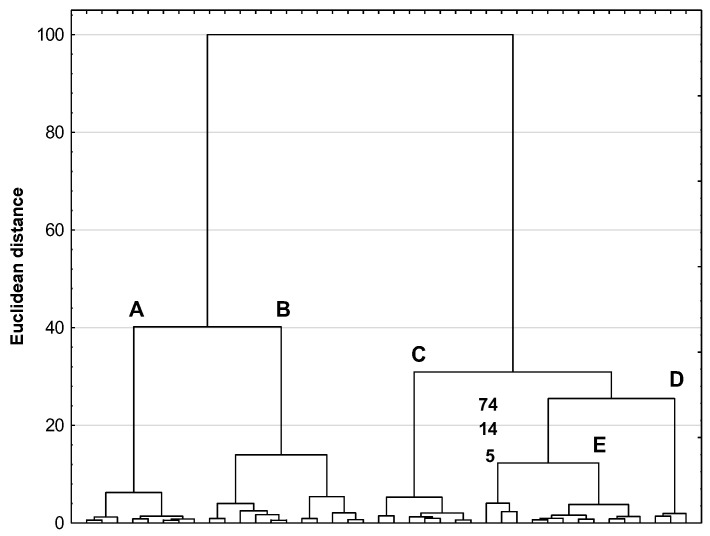
HCA dendrogram for the medicinal herbs and spices originating from the same species of medicinal plants. Clusters A, B, C, D and E group the same medicinal herbs and spices as the PCA score scatterplot shown in Figure 5.

HCA, a second technique of multivariate analysis, was also used to interpret the experimental data obtained. HCA calculations boil down to determining the distance between objects or variables in a multidimensional space. On this basis, the study samples are grouped into specific clusters, with two samples being more dissimilar the greater the distance between them. Graphically presented in Figure 7, the results of the HCA calculation show that the medicinal herbs and spices examined form five clusters, A, B, C, D, and E, with identical composition (medicinal herbs and spices) as in the PCA calculation. In addition, also in this case, three spices, 5, 14 and 74, form a separate cluster. This indicates a very high correspondence between the PCA and HCA results, which confirm some differences in the phenolic profiles and antioxidant properties of the five medicinal plant species studied. This is reflected in the five distinct clusters in the PCA and HCA plots.

## 4. Conclusions

The study showed that medicinal herbs and spices from plants belonging to the Lamiaceae family are a much richer source of phenolic compounds and exhibit stronger antioxidant properties than raw materials from plants in the Apiaceae family. Strong correlations between antioxidant activity determined by DPPH, ABTS and FRAP assays and the total content of phenolic compounds confirm that phenolic compounds contribute significantly to the antioxidant properties of medicinal herbs and spices. Multivariate statistical techniques, PCA and HCA confirmed that the studied plant materials differ in terms of TPC, FC, TAC and FIC values, with no significant differences in terms of phenolic and antioxidant profiles between medicinal herbs and spices from the same plant species, viz. oregano (*Origanum vulgare*), common thyme (*Thymus vulgaris*), rosemary (*Rosmarinus officinalis*), caraway (*Carum carvi*) and lovage (*Levisticum officinale*). Overall, chemometric techniques make it possible to identify plant species distinguished by their high content of phenolic compounds and high antioxidant activity.

## Figures and Tables

**Table 1:** General characteristics of the studied medicinal herbs and spices.

Sample Numbers	Raw Materials	Herbs Spices	Forms	Plant Species	Plant Families
1	Oregano herbs	herb	cut leaves	Oregano *Origanum vulgare* L	Lamiceaae
2–8	Oregano	spice	cut leaves and flowers		
9–11	Thyme herbs	herb	cut herbs	Common thyme *Thymus vulgaris* L.	
12–15	Thyme	spice	cut herbs		
16–19	Rosemary leaves	herb	cut leaves	Rosemary *Rosmarinus officinalis* L.	
20–22	Rosemary	spice	leaves		
23–29	Lemon balm leaves	herb	leaves	Lemon balm *Melissa officinalis* L.	
30–35	Peppermint leaves	herb	leaves	Peppermint *Mentha piperita* L.	
36–41	Sage leaves	herb	leaves	Common sage *Salvia officinalis* L.	
42–44	Savory	spice	cut herbs	Summer savory *Satureja hortensis* L.	
45–46	Hyssop	spice	cut herbs	Hyssop *Hyssopus officinalis* L.	
47–52	Basil	spice	cut leaves	Basil *Ocimum basilicum* L.	
53–60	Marjoram	spice	cut herbs	Marjoram *Origanum majorana* L.	
61–63	Caraway seeds	herb	whole seeds	Caraway *Carum carvi* L.	Apiceae
64–68	Caraway	spice	whole seeds		
69–73	Lovage root	herb	crushed root	Lovage *Levisticum officinale* Koch	
74–78	Lovage	spice	cut leaves		
79–82	Angelica root	herb	crushed root	Garden angelica *Archangelica officinalis* Hoffm.	
83–87	Tarragon	spice	cut herbs	Mugwort tarragon *Artemisia dracunculus* L.	Asteraceae

**Table 2:** Calibration curves and validation parameters of the methods used for the determination of TPC, FC and TAC.

Calibration Curves	TPC	FC	ABTS	FRAP
	Gallic Acid µg/mL (*n* = 7)	Rutin µg/mL (*n* = 6)	Trolox µmol/mL (*n* = 7)	Iron(II) Sulfate µmol/L (*n* = 6)
Range	1.0–8.0	4.0–28.0	0.1–1.0	2.5–40
Slope (a)	0.1161	0.03089	92.8087	0.0210
∆a (t_α,f_ · S_a_)	0.0021	0.0015	4.7867	0.0001
S_a_ (SD of the slope)	0.0008	0.0005	1.9561	0.00005
Intercept (b)	−0.0033	−0.0282	0.3651	−0.0062
∆b (t_α,f_ · S_b_)	0.0103	0.0273	0.8933	0.0030
S_b_ (SD of the intercept)	0.0040	0.0098	1.2521	0.0011
R^2^ (%)	99.97	99.84	99.73	99.99
S_xy_ (residual SD)	0.0052	0.0116	1.6245	0.0017
LOD (3,3 · S_xy_/a)	0.1477	1.2412	0.0578	0.2726
LOQ (10 · S_xy_/a)	0.4475	3.7611	0.1750	0.8262
Recovery and precision				
Concentration of standard	4.0	20.00	0.40	20.0
Determined concentration	3.91	20.25	0.39	19.90
Recovery (%)	97.75	101.24	97.75	99.51
Intra-day CV (%)	4.66	2.26	1.89	3.44

Regression equation y = ac + b, where c—concentration, y—absorbance, t_α,f_—t-Student’s coefficient for a given degree of freedom (f) and significance level (α), SD—standard deviation, CV—coefficient of variation.

**Table 3:** Results of the determination of total content of phenolic compounds (TPCs); flavonoid content (FC); total antioxidant capacity according to DPPH, ABTS and FRAP tests; and ferrous-ion-chelating capacity (FIC) for the studied medicinal herbs and spices.

Sample Numbers	Raw Materials	TPC mg GAE/g	FC mg RUT/g	TAC_DPPH_ %	TAC_ABTS_ mmol TEAC/g	TAC_FRAP_ mmole Fe(II)/g	FIC %
1	Oregano herbs	81.77–84.27 82.66 (82.31)	23.65–26.89 25.40 (25.53)	51.08–64.38 57.67 (57.61)	5.63–6.22 5.94 (5.96)	1.30–1.43 1.36 (1.36)	51.16–58.45 55.08 (55.36)
2–8	Oregano	31.38–97.77 54.30 (50.32)	8.53–36.91 18.82 (17.71)	30.26–77.48 47.94 (40.80)	2.25–7.66 4.00 (3.44)	0.46–1.96 0.87 (0.61)	20.06–71.70 51.62 (58.73)
9–11	Thyme herbs	41.38–59.94 49.39 (48.69)	17.92–23.84 21.16 (21.11)	32.91–44.32 39.03 (40.00)	3.19–4.45 3.68 (3.69)	0.60–1.02 0.81 (0.83)	54.21–68.52 60.84 (61.14)
12–15	Thyme	27.56–55.75 46.85 (51.51)	10.72–25.12 19.76 (21.20)	27.74–53.44 42.83 (44.80)	1.78–4.15 2.89 (2.88)	0.42–2.64 1.44 (1.42)	48.78–64.64 58.19 (59.89)
16–19	Rosemary leaves	39.49–50.63 47.07 (48.36)	13.78–16.97 15.84 (16.03)	24.05–50.72 39.58 (39.37)	3.10–4.47 3.74 (3.79)	0.64–0.96 0.77 (0.75)	20.39–45.51 31.55 (30.58)
20–22	Rosemary	45.51–59.90 52.50 (52.32)	13.63–19.42 17.19 (17.90)	34.69–42.03 38.50 (39.18)	3.78–4.63 4.04 (3.98)	0.52–0.80 0.67 (0.65)	30.44–49.18 39.75 (39.95)
23–29	Lemon balm leaves	60.29–100.14 87.54 (88.72)	13.64–23.43 18.73 (18.68)	55.05–92.87 74.89 (76.65)	3.37–7.81 5.99 (6.18)	1.07–2.56 1.67 (1.62)	51.37–73.96 61.07 (61.40)
30–35	Peppermint leaves	37.06–74.99 54.75 (46.75)	13.47–40.04 27.18 (26.96)	32.53–66.28 46.70 (46.31)	2.07–5.38 3.72 (3.67)	0.54–1.41 0.91 (0.88)	46.84–68.42 60.78 (62.66)
36–41	Sage leaves	17.39–41.22 33.84 (35.02)	6.32–26.77 18.61 (20.21)	17.46–41.74 30.72 (32.72)	1.47–3.37 2.62 (2.85)	0.37–0.73 0.54 (0.54)	56.84–73.56 67.06 (68.69)
42–44	Savory	20.89–45.14 35.97 (40.77)	12.98–23.16 17.23 (17.54)	22.48–54.01 34.36 (34.90)	1.16–3.45 2.00 (1.78)	0.30–0.65 0.53 (0.63)	70.74–80.59 76.26 (76.40)
45–46	Hyssop	40.89–51.55 45.19 (44.96)	5.16–9.12 7.80 (8.46)	33.22–56.52 43.80 (43.77)	2.50–3.82 3.18 (3.20)	0.61–1.03 0.84 (0.80)	43.79–56.26 50.73 (51.47)
47–52	Basil	25.72–39.85 33.65 (33.01)	8.12–15.87 10.92 (11.13)	24.69–37.23 30.20 (30.23)	1.25–3.59 2.37 (2.31)	0.30–0.68 0.49 (0.49)	50.10–73.45 62.55 (61.43)
53–60	Marjoram	33.64–68.02 49.08 (47.17)	13.85–24.22 19.21 (19.32)	26.42–58.16 39.91 (40.46)	2.75–6.47 4.38 (4.53)	0.45–0.81 0.63 (0.61)	48.00–68.52 58.65 (60.18)
61–63	Caraway seeds	7.04–10.61 8.52 (8.36)	4.88–7.09 5.97 (5.77)	11.77–19.67 15.72 (15.58)	0.59–1.03 0.85 (0.92)	0.06–0.09 0.08 (0.08)	40.92–62.49 54.72 (58.13)
64–68	Caraway	5.96–11.72 8.06 (7.97)	3.22–6.19 4.70 (4.63)	11.52–16.50 13.75 (13.28)	0.17–0.99 0.70 (0.73)	0.04–0.10 0.07 (0.06)	41.66–65.26 54.60 (53.65)
69–73	Lovage root	3.70–7.90 6.28 (6.47)	1.55–5.57 3.46 (3.88)	10.72–16.56 14.18 (14.69)	0.55–1.28 0.88 (0.90)	0.05–0.08 0.07 (0.06)	4.95–35.79 16.81 (15.95)
74–78	Lovage	8.39–26.46 16.71 (16.11)	7.86–19.56 14.23 (15.02)	11.3–22.36 15.40 (14.39)	0.80–2.23 1.34 (1.27)	0.08–0.28 0.17 (0.15)	16.31–72.95 41.17 (39.26)
79–82	Angelica root	5.00–7.14 5.66 (5.41)	2.24–4.07 2.82 (2.71)	10.91–17.00 13.47 (13.57)	0.43–2.24 0.83 (0.77)	0.03–0.08 0.06 (0.06)	28.64–70.95 51.27 (54.13)
83–87	Tarragon	19.82–47.21 32.35 (29.63)	12.50–29.00 20.24 (18.74)	17.80–41.67 23.51 (27.03)	1.58–4.06 2.48 (2.21)	0.19–0.62 0.36 (0.29)	32.20–78.11 58.74 (57.71)

The results of the determinations are given per dry mass of raw material (d.m.). For each raw material, the range of values, arithmetic mean and median (in parentheses) are given.

## Data Availability

The data are contained within this article.
